# Capitonnage Versus Non‐Capitonnage in Pediatric Pulmonary Hydatid Disease: A Systematic Review and Meta‐Analysis

**DOI:** 10.1002/hsr2.70235

**Published:** 2024-12-04

**Authors:** Mohammad Javad Boozhmehrani, Seyed Sobhan Bahreiny, Mohammad Navid Bastani, Mahdi Amraei, Zahra Mansouri, Razieh Kazemzadeh, Majid Farhadi, Akbar Hoseinnejad, Ali Pirsadeghi, Zahra Asadi, Afshin Bighamian, Gilda Eslami

**Affiliations:** ^1^ Department of Medical Parasitology, Faculty of Medicine Jundishapur University of Medical Sciences Ahvaz Iran; ^2^ Student Research Committee Ahvaz Jundishapur University of Medical Sciences Ahvaz Iran; ^3^ USERN Office Jundishapur University of Medical Sciences Ahvaz Iran; ^4^ Environmental Health Research Center Lorestan University of Medical Sciences Khorramabad Iran; ^5^ Clinical Research Development Unit, Golestan Hospital Ahvaz Jundishapur University of Medical Sciences Ahvaz Iran; ^6^ Department of Parasitology and Mycology School of Medicine, Isfahan University of Medical Sciences Isfahan Iran

**Keywords:** capitonnage, Echinococcus granulosus, pulmonary hydatid, surgery

## Abstract

**Background and Aim:**

Pulmonary hydatid disease, caused by *Echinococcus granulosus*, presents significant clinical challenges, particularly in pediatric populations. Surgical intervention remains the gold standard for treatment, with various techniques employed, including capitonnage and non‐capitonnage methods. This systematic review and meta‐analysis evaluates the efficacy and safety of capitonnage compared to non‐capitonnage techniques in children.

**Methods:**

This systematic review and meta‐analysis followed the PRISMA guidelines to ensure methodological rigor. A comprehensive literature search was conducted across PubMed, Web of Science, and Scopus databases to identify relevant studies. To assess pooled event rates and corresponding 95% confidence intervals for both complications and cure rates, we employed a random‐effects model, allowing for variability among study populations. All statistical analyses were conducted using Comprehensive Meta‐Analysis software (version 3.7).

**Results:**

Thirteen studies met the established inclusion criteria for analysis. The overall complication rate was 46%, with significantly lower rates in the capitonnage group (24%) compared to the non‐capitonnage group (58%). The cure rate was higher in the capitonnage group (83.5%) than in the non‐capitonnage group (65.2%). Meta‐regression analysis indicated that complication rates were influenced by cyst diameter, study publication date, mean age, and type of surgery.

**Conclusion:**

The findings suggest that capitonnage is associated with better outcomes in terms of lower complication rates and higher cure rates. This evidence supports the use of capitonnage as a preferred surgical technique for managing pulmonary hydatid disease in children. Further research is recommended to explore the long‐term outcomes and potential benefits of combining surgical and pharmacological treatments.

## 1 Introduction

Pulmonary hydatid cysts remain prevalent in regions such as South Africa, South America, the Middle East, India, Australia, and the Mediterranean region. Factors contributing to the high prevalence of *Echinococcus granulosus* infection in rural areas include farming livestock (the intermediate host) and close contact with dogs (the main reservoir of infection), often associated with tribal lifestyles [1, 2].

*E. granulosus* can form hydatid cysts in various tissues, including the brain, heart, lungs, liver, and spleen [3]. In children, pulmonary hydatid cysts are more common, whereas in adults, they primarily occur in the liver [4].

The lung's elasticity and negative chest pressure make it highly susceptible to hydatid cyst growth, facilitated by larval dissemination through the hepatic sinusoids [5]. Pulmonary hydatid cysts may lead to increased mediastinal pressure and bronchial tree rupture, with potential complications such as lung lesions and cyst rupture into the pleural cavity. Common symptoms include respiratory distress, coughing, chest pain, fever, hemoptysis, and hydatid vomiting [6].

According to a World Health Organization (WHO) report, approximately one million individuals worldwide are infected with *E. granulosus* annually. Of these, hydatid cysts form in the lungs in about 250,000 cases (25%) [7, 8].

Several surgical methods exist, including posterolateral thoracotomy, open enucleation, pericystectomy, cystotomy with or without capitonnage, segmental resection, bronchial fistula closure, segmentectomy, or lobectomy. The choice of technique depends on the patient's condition and the surgeon's discretion [9, 10, 11, 12, 13, 14].

The objective of this study is to evaluate the efficacy and safety of capitonnage as a preferred surgical technique for treating pulmonary hydatid cysts in children.

## 2 Methods

### 2.1 Protocol and Registration

To ensure transparency and maintain a rigorous review process, the systematic review and meta‐analysis were conducted according to the Preferred Reporting Items for Systematic Reviews and Meta‐Analyses (PRISMA) guidelines. In addition, the systematic review protocol was registered in *PROSPERO*, a specialized international database dedicated to the prospective registration of systematic reviews, under the registration code CRD42023429957 [15, 16].

### 2.2 Eligibility Criteria

We reviewed and compared all studies published between January 2000 and February 2023 on the effect of different surgical techniques, especially capitonnage, on children with pulmonary hydatid disease. The inclusion criteria were carefully selected to ensure a targeted investigation into the treatment of pediatric patients with this condition. Specifically, eligible studies were required to concentrate on pediatric populations afflicted with pulmonary hydatid disease and to employ appropriate study designs, including both comparative and retrospective methodologies. Additionally, studies were only considered if they provided sufficient data for thorough analysis. Moreover, to enhance the robustness of our findings, we further refined our inclusion criteria to encompass studies with clear reporting on outcomes relevant to the efficacy of different surgical interventions.

Conversely, the exclusion criteria were designed to filter out articles that did not align with the objectives of our study. Excluded articles comprised review articles, case reports, conference abstracts, and studies not primarily focused on pulmonary cysts. By delineating these criteria, our intent was to ensure a focused and rigorous scientific evaluation of the role of capitonnage in the treatment of pulmonary hydatid disease in children.

### 2.3 Literature Search

The literature search was conducted using the title and abstract fields in three databases: PubMed, Web of Science, and Scopus. The search terms used included various keywords related to hydatid disease and echinococcosis, such as “Hydatid*,” “Echinococcosis,” “Echinococcoses,” “*Echinococcus* Infection,” “*Echinococcus* Infections,” “Infection, *Echinococcus,*” “Cystic Echinococcosis,” “Cystic Echinococcoses,” “Echinococcoses, Cystic,” “Echinococcosis, Cystic,” “Hydatidosis,” “Hydatidoses,” “Cysts, Hydatid,” “Cyst, Hydatid,” “Hydatid Cysts,” “Hydatid Cyst,” “Hydatid Disease,” “Hydatid Diseases,” “*Echinococcus Granulosus* Infection,” “*Echinococcus Granulosus* Infections,” “*Granulosus* Infection, *Echinococcus*”, “*Granulosus* Infections, *Echinococcus*,” “Infection, *Echinococcus Granulosus*,” “Infections, *Echinococcus Granulosus*,” “Echinococcosis, Pulmonary,” “Cysts, Pulmonary Hydatid,” “Echinococcoses, Pulmonary,” “Hydatid Cyst, Pulmonary,” “Hydatid Cysts, Pulmonary,” “Pulmonary Hydatidosis,” “Hydatidosis, Pulmonary,” “Pulmonary Echinococcoses,” “Pulmonary Echinococcosis,” “Pulmonary Hydatid Cyst,” “Pulmonary Hydatid Cysts,” “Pulmonary Hydatidoses,” “Cyst, Pulmonary Hydatid,” “Hydatidoses, Pulmonary,” “Capitonnage,” “Cystotomy,” “Cystotomies,” “Vesicotomy,” “Vesicotomies,” “General Surgery,” “Surgery, General,” “Surgery,” “Operative therapy,” “Invasive procedures,” “Operative procedures,” “Operations,” “Perioperative procedures,” “Intraoperative procedures,” “Peroperative procedures,” “Preoperative procedures,” “Cyst rupture,” “Surgical Procedures, Operative,” “Operative Procedures,” “Operative Procedure,” “Procedure, Operative,” “Procedures, Operative,” “Surgical Procedure, Operative,” “Operative Surgical Procedures,” “Procedure, Operative Surgical,” “Procedures, Operative Surgical,” “Surgical Procedures,” “Procedure, Surgical,” “Procedures, Surgical,” “Surgical Procedure,” “Operative Surgical Procedure,” “Surgery, Ghost,” “Ghost Surgery,” “Lung,” “Lungs,” “Pulmonary,” “Child*,” “pediatric*.”

These keywords were logically combined using “AND” and “OR” conjunctions to refine the search results effectively. In addition, references to related articles were searched manually, and the search terms were supplemented by searching the available gray literature. All retrieved records were imported into EndNote 20 software, and duplicate studies were removed during the screening process.

### 2.4 Study Selection

Two independent reviewers, A.H. and M.J.B., meticulously scanned the available literature and assessed the eligibility criteria for the study. Reviewer A.H. conducted the initial screening, while M.J.B. independently reviewed the selected studies. In cases where there were disagreements between the reviewers, collaborative discussions were held to resolve them. These discussions involved referring back to the predefined inclusion/exclusion criteria and seeking additional information as needed. To further enhance the selection process and ensure consensus, a third reviewer, S.B., highly specialized in statistical analysis, was involved. S.B. provided additional insights and perspectives, particularly leveraging their expertize in statistical methods to evaluate study design and data analysis. Collaborative discussions among the reviewers, with S.B.'s statistical expertize, helped ensure a rigorous and comprehensive study selection process for the review article.

### 2.5 Data Extraction

The data extraction process for the review article on the effect of capitonnage in the treatment of children with pulmonary hydatid disease involved a systematic approach using a predetermined checklist. The following data items were extracted from each included study: Study Name (Author, Year), providing the complete citation including the name of the author(s) and the year of publication to uniquely identify each study; Country, indicating where the study was conducted to provide geographical context and potential variations in clinical practices; Study Design, detailing the specific study design employed, such as randomized controlled trials, prospective cohort studies, or retrospective comparative studies, enabling assessment of the quality of evidence; Capitonnage (Yes/No), specifying whether capitonnage was performed as a surgical technique in the study, indicating the presence or absence of this intervention; Total Number of Patients, revealing the total number of patients included in the study to provide an understanding of the sample size; Hospital Stay (Days), reflecting the duration of hospital stay for the patients, which is indicative of the postoperative recovery period; Duration of Follow‐up (Months), indicating the length of time for which patients were followed up after the surgical intervention to assess long‐term outcomes; Cure (Dichotomous–Event Rate), representing the cure rate as a dichotomous outcome to indicate the proportion of patients who achieved a successful outcome; Complications (%), showing the percentage of patients who experienced complications or adverse events during the treatment or follow‐up period; Cyst Diameter (cm) Mean, providing the mean diameter of the pulmonary hydatid cysts measured in centimeters to give information on the size of the cysts; Age (Years), detailing the average age of the patients included in the study to offer insights into the age distribution and potential age‐related factors.

Data extraction involved carefully reviewing each included study and recording the relevant information based on the predetermined checklist, adhering to the study extraction design outlined in our previous research [17, 18, 19]. This systematic approach ensured accurate and consistent data collection, enabling a comprehensive analysis of the effect of capitonnage in the treatment of children with pulmonary hydatid disease across the selected studies. (Table 1).

**Table 1:** Characteristics of the included studies.

Author name	Country	Study Design	Capitonnage or not (yes or no)	Total number of patients	Hospital stay (day)	Duration of follow‐up (mo.)	Curedichotomous (event rate)	Complication (%)	Cyst diameter (cm)Mean	Age (yr.)
Karavdic 2011 [4]	Bosnia	Retrospective study	No	72	N/A	72	91/63	5/56	N/A	10.36
Cevik 2013 [20]	Turkey	Retrospective study	Yes	120	7.27	11.3	84.9	15.1	3.89	10.15 ± 3.3
Balci A 2002 [21]	Turkey	Retrospective study	Yes	63	4	19.3	87.61	47.9	> 10	12.3
Balci B 2002 [21]	Turkey	Retrospective study	No	63	4	19.3	47.7	76.4	> 10	12.3
Amine 2014 [22]	Tunisia	Retrospective study	Yes	25	5	48	96	4	< 5	8
Aydin Cangir 2013 [23]	Turkey	Retrospective study	Yes	42	7.2	39	95	4.76	6.2	5.2 ± 1.3
Kurkcuoglu 2004 [24]	Turkey	Retrospective study	Yes	102	9.3	60	N/A	9.8	10	10.2
Haberal 2018 [10]	Turkey	Retrospective study	Yes	25	7	12	84	16	< 7	10/5
Kabiri 2019 [12]	Morocco	Retrospective study	Yes	19	13/5	24	89/5	10/5	n	9/4
Ksia A. 2019 [25]	Tunisia	Retrospective study	Yes	136	5/6	N/A	69	31	10	7/8
Ksia B. 2019 [25]	Tunisia	Retrospective study	No	136	6/7	N/A	35	65	10	8/1
Ngcobo A. 2020 [14]	South Africa	Retrospective study	Yes	48	5	N/A	75	25	7	6/5
Ngcobo B. 2020 [14]	South Africa	Retrospective study	No	48	7	N/A	40	60	7	6/5
Khalfallah 2021 [26]	Tunisia	Retrospective study	Yes	105	7	3	87.7	12.6	12.7	10.5 ± 3
He 2022 [11]	China	Retrospective study	No	12	8	36	91.7	8/3	12.8	8.7
Kocaman 2022 [13]	Turkey	Retrospective study	Yes	94	8.76 ± 4.80	32.4	9.00	18/1	˂ 10	8.95 ± 3.88

*Note:* “N/A” stands for “not available”.

### 2.6 Data Analysis and Evidence Synthesis

Pooled event rates and 95% confidence intervals (CIs) were estimated for complications and cure using a random‐effects model [27]. We tested for heterogeneity operating two heterogeneity testing methods, namely the Cochran Q test (where the *p* < 0.05 was considered significant) and the I2 index. I2 values of 75%, 50%, and 25% corresponded to high, moderate, and low levels of heterogeneity, respectively [28]. The publication bias was revealed using a funnel plot and assessed with Begg's and Egger's weighted regression tests [29]. In addition, meta‐regression analysis was performed to test the association between complication rate and cyst diameter, study publication date, mean age, Total sample size, and type of operation. All analyses were performed using Comprehensive Meta‐analysis (version 3.7).

## 3 Results

### 3.1 Characteristics of the Included Studies

**Figure 1:**
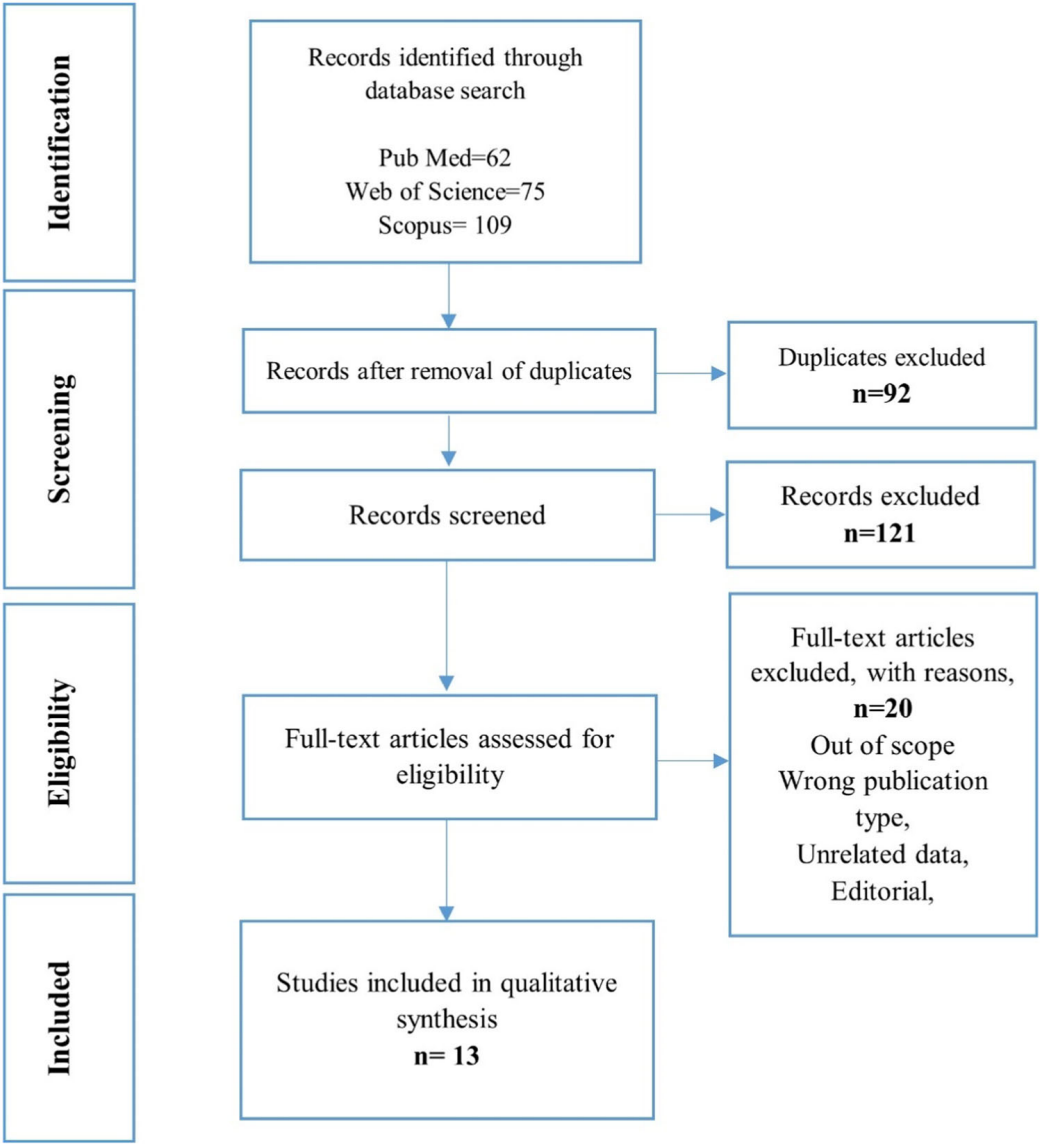
PRISMA flow chart showing the study selection process.

According to the initial search strategy, 246 records were retrieved. 92 records were removed due to duplicate data and 121 articles were excluded by reading the titles and abstracts. In the next stage of document screening, the remaining articles were evaluated by full text. Finally, 13 articles (containing 16 datasets suitable for meta‐analysis) were identified to investigate the difference between Non‐Capitonnage and Capitonnage surgical approaches for pulmonary hydatid cysts. Figure 1 shows the screening process explained.

### 3.2 Quantitative Synthesis

#### 3.2.1 Event Rates

**Figure 2:**
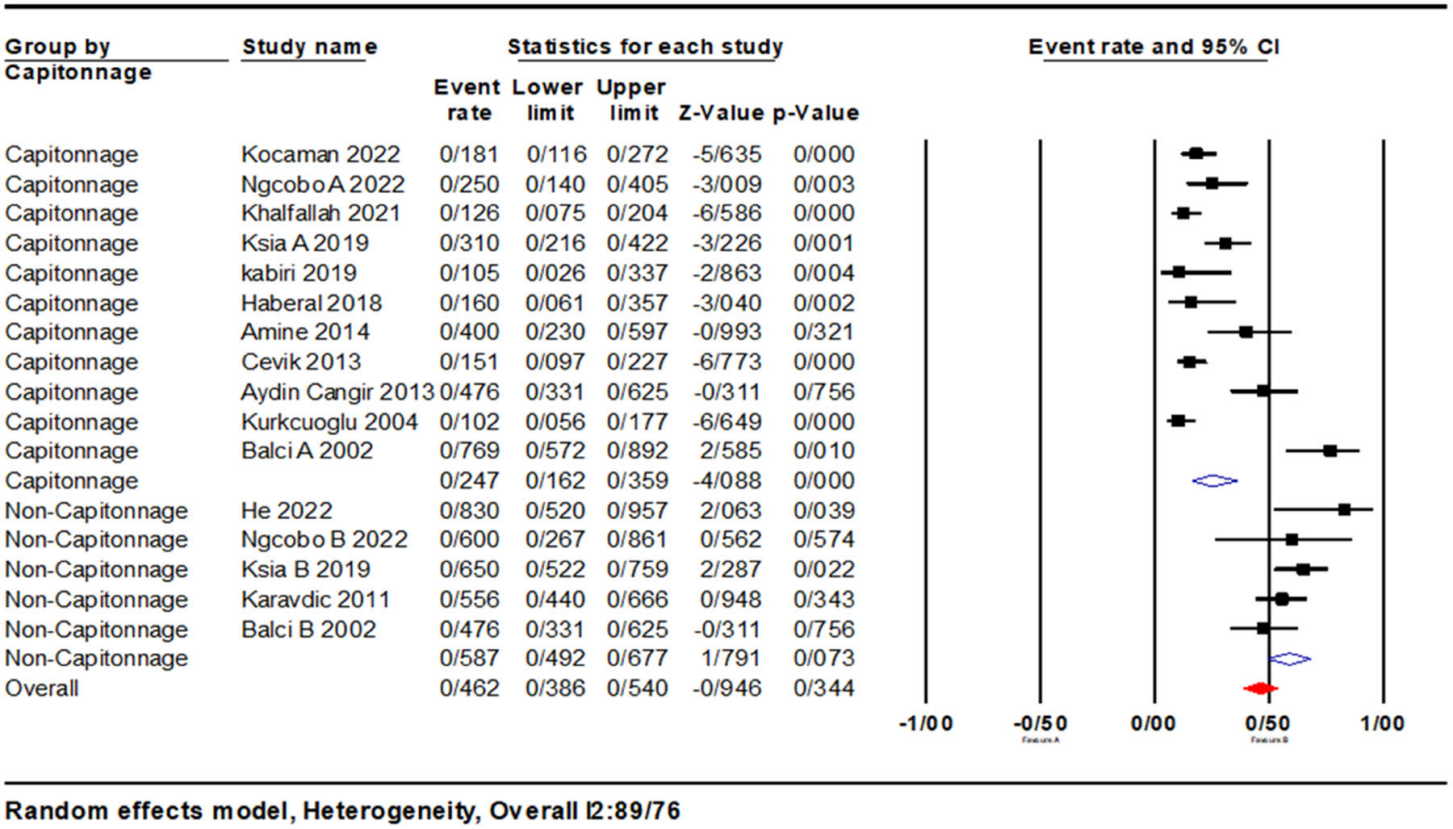
Forest plots of Event rates of outcomes (complications), between Capitonnage and non‐Capitonnage surgeries.

The overall complication rate was 46% (95% CI: 0.38–0.54). Furthermore, based on subgroup studies, the complication rate in the capitonnage group was 24% (95% CI: 0.10–0.35). In the non‐capitonnage group, the rate of complications was estimated to be higher at 58% (95% CI: 0.49–0.67) (Figure 2).

**Figure 3:**
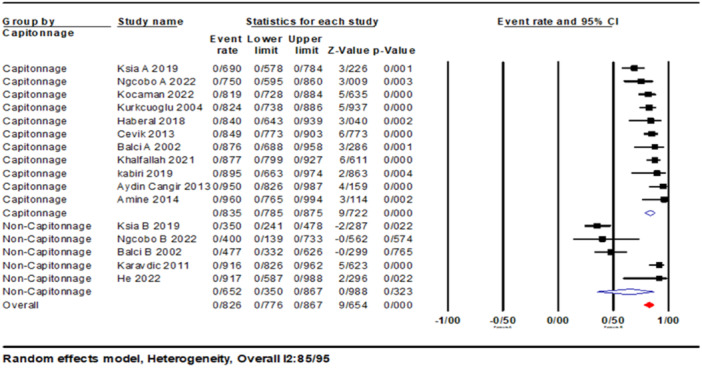
Forest plots of event rates of outcomes (cure), between capitonnage and non‐capitonnage surgeries.

Additionally, the cure rate of the surgical groups was estimated. The overall cure rate was 82.6% (95% CI: 0.77–0.86). Based on subgroup studies, the cure rate in the capitonnage group was 83.5% (95% CI: 0.78–0.87). In the non‐capitonnage group, the cure rate was 65.2% (95% CI: 0.35–0.86) (Figure 3).

#### 3.2.2 Meta‐Regression

**Figure 4:**
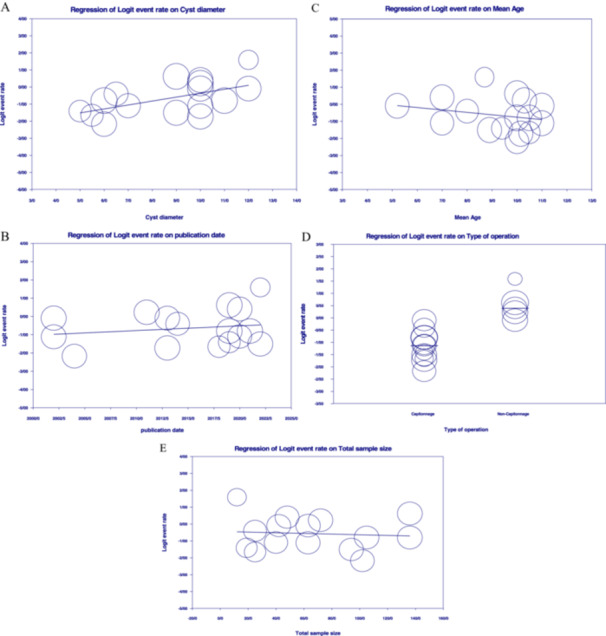
Meta‐regression of Event rate of Complications in Surgical management of pulmonary hydatid cysts. (A) Based on cyst diameter, (B) Based on study publication date, (C) Based on mean age date, (D) Based on type of operation (larger circles indicate larger sample size), and (E) Based on Total sample size. (A) Based on Cyst diameter. (B) Based on study publication date. (C) Based on Mean Age date. (D) Based on type of operation. (E) Based on total sample size.

Due to significant differences in baseline characteristics among the included studies, there is a substantial limitation in interpreting the pooled effect estimates derived from these studies. A meta‐regression analysis was performed to determine the observed differences in the rate of surgical complications between the two surgical groups (Figure 4).

Meta‐regression showed that participants' cyst diameter was independent of the magnitude of the event rate (meta‐regression coefficient: 0.103; 95% CI: −0.14 to 0.35, *p* = 0.41). The study by Karavdic and Balci [4, 21]. was excluded because the cyst diameter of participants was not available. Additionally, the publication date (meta‐regression coefficient: 0.065; 95% CI: −0.04 to 0.17, *p* = 0.24) was not predictive of increased complication rates. The variables that affected the outcome were the type of operation and mean age. The meta‐regression analyses showed that mean age was associated with complications (meta‐regression coefficient: −0.377; 95% CI: −0.64 to −0.11, *p* = 0.005). Furthermore, these meta‐regression results confirmed the earlier results of the subgroup meta‐analysis by type of operation (meta‐regression coefficient: 2.106; 95% CI: 1.08 to 3.12, *p* < 0.001). Meta‐regression analysis revealed that the rate of surgical complications was not significantly influenced by the total sample size across studies, showing a negative but nonsignificant correlation (meta‐regression coefficient: −0.002; 95% CI: −0.013 to 0.10, *p* = 0.74). This suggests that differences in sample size among studies did not substantially alter the complication rate between the two techniques.

### 3.3 Sensitivity Analysis and Publication Bias

**Figure 5:**
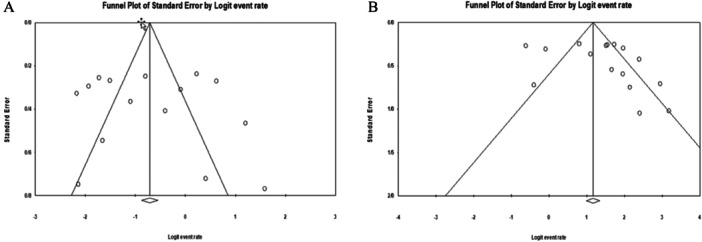
Publication bias in the studies for the event rate of complications (A) and cure (B).

A sensitivity analysis was performed by removing each study and repeating the analysis. There was no significant change in the pooled effects, indicating the stability and reliability of the meta‐analysis results. Begg's and Egger's tests were used to assess the publication bias of the included studies. The funnel plot showed no apparent asymmetry in any genetic models, which showed no potential publication bias (Figure 5). The statistical results of Egger's and Begg's tests also suggest no publication bias between studies (*p* < 0.05).

## 4 Discussion

The surgical management of lung hydatid cysts involves various methods depending on factors such as cyst size, location, patient age, and available treatment facilities. This systematic review aims to assess the use of the capitonnage technique in lung hydatid cyst surgery. While some studies have advocated for capitonnage as a suitable treatment method [22, 25, 30, 31], conflicting opinions have also been reported [9, 32, 33]. Given the inconsistent results, this review conducted a comprehensive analysis to determine whether capitonnage is a more effective approach for treating hydatid lung cysts in children compared to other methods. Notably, this is the first systematic review to rigorously evaluate the application of the capitonnage technique specifically for treating pulmonary hydatid cysts in children, along with its associated complications. The review identified and analyzed 13 eligible articles discussing surgical treatment of pleural hydatid cysts, considering factors such as capitonnage‐related complications, comparison with non‐capitonnage techniques, recovery rates, cyst diameter, and patient age.

The overall treatment rate for hydatid cyst surgery is reported to be 82.6% (regardless of the type of surgery). The recovery rate for cyst surgery using the capitonnage technique is 83.5%, with reported rates ranging from 69% (lowest) to 96% (highest) in the included studies [22, 25]. In contrast, the non‐capitonnage technique exhibits a recovery rate of 65.2%, with reported rates ranging from 35% (lowest) to 91% (highest) [11, 25]. These findings suggest that hydatid cyst surgery with the capitonnage technique yields better treatment outcomes. Furthermore, Cevik et al. [13] concluded that the combination of drug treatment and surgery is the most effective approach, employed in 90% of cases. Haberal et al. [10] reported a recurrence rate ranging from 2% to 25%, while no cases of recurrence or death were observed in their study population. However, it is noteworthy that capitonnage surgery is generally associated with a mortality rate between 0.5% and 4%. These results align with previous studies conducted by Şehitoğulları, Aldahmashi, and Lichter [34, 35, 36]. A comparative analysis of capitonnage and non‐capitonnage surgical procedures conducted by Akar et al. [37] revealed that the non‐capitonnage group had higher rates of pneumothorax (30% vs. 13.2%), emphysema, and residual cavity persistence (23.3% vs. 7.9%) compared to the capitonnage group. Capitonnage significantly reduces the occurrence of pneumothorax, emphysema, and postoperative residual cavities, resulting in decreased postoperative air leaks and hospitalization [25]. Moreover, Ngcobo et al.'s study [14] demonstrated a 75% success rate with no postoperative complications or recurrences observed after 1 year of capitonnage surgery.

### 4.1 The Overall Rate of Complications

This study investigated the rate of complications associated with different surgical techniques employed for the removal of lung hydatid cysts, aiming to provide a comprehensive analysis of the overall complications encountered. The findings reveal an overall complication rate of 46% attributed to the surgical procedures utilized in treating lung hydatid cysts. Notably, complications appear to be more prevalent in cases involving non‐capitonnage techniques. Specifically, the average rate of complications for the capitonnage technique is 24.7%, with the lowest recorded rate being 18.1% [24], whereas the highest rate of complications reaches 76.9% [38]. On the other hand, the average complication rate following non‐capitonnage techniques is 58.7%. The lowest rate of postoperative complications associated with the non‐capitonnage technique is reported at 47.6% [38], while the highest rate is observed at 83% [11].

Khalfallah et al. [26] conducted a study focusing on children with pulmonary hydatid cysts, particularly those with complex and giant cyst features. Among the 105 children included in the study, 13 experienced immediate postoperative complications, predominantly observed in cases involving complex and giant cysts necessitating extensive parenchymal resection. In Cevik et al.'s study [20], cough was identified as the most common symptom among patients with hydatid cysts, while anaphylactic shock occurred in 1.3% of patients. It has been posited by Ksia and Haberal [10, 22] that capitonnage represents a favorable technique for treating hydatid cysts in children due to its reduced pain and lower occurrence of short‐term and long‐term complications. Researchers also suggest that adults with hydatid cysts are less prone to experiencing anaphylactic shock compared to younger individuals, and pulmonary cysts are more likely to elicit anaphylactic shock compared to liver cysts [4, 20, 39].

In contrast, Karavdic and colleagues [40] argue that the non‐capitonnage surgical method offers superior treatment outcomes for patients with hydatid cysts, citing fewer complications post‐surgery, shorter operative durations, and reduced secretion drainage. In their study involving 72 pediatric patients treated with the non‐capitonnage method, only four minor postoperative complications were reported (two cases of surgical wound infections and two cases of long‐term parenchymal air leaks), all of which were effectively managed by the attending physician. On the other hand, Amine et al. [22] contend that the capitonnage surgical technique represents a superior approach for treating pulmonary hydatid cysts, as it effectively prevents the formation of emphysema and residual cavities.

### 4.2 Hydatid Cyst Diameter

Hydatid cyst diameter is a crucial factor influencing the outcomes of surgical intervention when the cyst invades the lung. Two possible scenarios can arise [1]: the cyst perforates into the bronchiole, prompting repair of the residual cavity and subsequent resolution of the disease [2]; the cyst expands due to the lung's elasticity. As the diameter of the hydatid cyst increases, the complications associated with surgical procedures also tend to escalate. The Cevik study demonstrated that patients with liver hydatid cysts tended to be older compared to those with lung hydatid cysts, likely due to the ease with which symptoms manifest in the lung tissue, which is more compressible, leading to increased cyst growth. Cysts in the lung, owing to reduced lung elasticity, may delay symptom onset. However, this study (Cevik) indicated that the cyst diameter has no impact on treatment outcomes [20].

Ksia et al. [25], in their investigation, concluded that the capitonnage technique is suitable for small cysts measuring less than 5 cm, as controlling the fistula requires only a single stitch. Conversely, capitonnage surgery poses challenges for larger cysts due to continuous air leakage. Haberal et al. [10] reported a higher incidence of pulmonary involvement in children, often attributable to delayed diagnosis, resulting in the identification of larger cysts. In their study, 5 out of 12 patients had cysts equal to or larger than 5 cm in size. The diameter of the lung cyst plays a pivotal role in its propensity to rupture during surgery. Many physicians and researchers hold the belief that lung cysts with a diameter exceeding 3 mm are likely to rupture [41, 42, 43]. However, Kuzucu et al. [44] contended that there is no correlation between intraoperative rupture of lung cysts and their size. According to the WHO recommendations, capitonnage surgery is more effective for hydatid cysts with diameters less than 5 cm compared to those exceeding 10 cm [45]. This viewpoint is also shared by Cevik et al. and Aydogdu et al. [20, 46]. Karaoglanoglu and Salih [31, 47] argued that giant cysts are associated with a higher incidence of postoperative complications, such as prolonged air leakage and atelectasis, as well as a longer hospital stay.

In conclusion, this meta‐analysis suggests that capitonnage may offer notable advantages over non‐capitonnage in pediatric patients undergoing surgery for pulmonary hydatid cysts. The lower complication rates and higher cure rates associated with capitonnage underscore its potential as a preferred approach, particularly for younger patients or cases involving small to moderately sized cysts. However, the choice of surgical technique should also consider individual patient factors, including cyst characteristics, surgeon expertize, and institutional capabilities. While non‐capitonnage may be appropriate in cases requiring shorter operative time or where cysts are anatomically challenging, capitonnage appears generally superior in minimizing postoperative complications like pneumothorax and emphysema. Physicians are encouraged to evaluate both techniques critically, balancing the potential benefits of capitonnage against the specific needs of each case to optimize patient outcomes. Further studies with larger sample sizes and prospective designs are warranted to strengthen these recommendations and refine surgical guidelines for this patient population.

## 5 Limitations

This study provides valuable insights through a comprehensive meta‐analysis comparing capitonnage and non‐capitonnage techniques in pediatric patients, along with subgroup analyses that help identify factors influencing outcomes. Nonetheless, it has several limitations. First, many of the included studies are retrospective, which may introduce bias. Additionally, some studies have small sample sizes, potentially affecting the generalizability of the results. Methodological differences and variations in patient characteristics across studies may also hinder direct comparability. Furthermore, limited geographic representation restricts the applicability of findings to other settings. There is also a possibility of publication bias, and the lack of long‐term follow‐up data limits the ability to assess the enduring efficacy and potential complications of these surgical methods.

## Author Contributions

**Mohammad Javad Boozhmehrani:** methodology, conceptualization, supervision, data curation, project administration, writing–review and editing, writing–original draft, visualization, resources. **Seyed Sobhan Bahreiny:** software, data curation, formal analysis, writing–original draft, methodology, investigation. **Mohammad Navid Bastani:** writing–original draft, project administration, supervision. **Mahdi Amraei:** methodology, software, data curation, investigation, visualization. **Zahra Mansouri:** methodology, software, data curation, investigation, visualization. **Razieh Kazemzadeh:** writing–original draft. **Majid Farhadi:** writing–original draft. **Akbar Hoseinnejad:** writing–original draft. **Ali Pirsadeghi:** writing–original draft. **Zahra Asadi:** writing–original draft. **Afshin Bighamian:** supervision, writing–review and editing. **Gilda Eslami:** writing–original draft, Writing–review and editing, supervision, methodology.

## Conflicts of Interest

The authors declare no conflict of interest. There are no financial or personal relationships that could inappropriately influence or bias the content of the research.

## Data Availability

The data that support the findings of this study are available from the corresponding author upon reasonable request.

## References

[1] J. E. Bennett , R. Dolin , and M. J. Blaser , Mandell, Douglas, and Bennett's Principles and Practice of Infectious Diseases E‐Book: 2‐Volume Set (Elsevier health sciences, 2019).

[2] S. Guska , Z. Cerimagić , and I. Pilav , “Conservative Surgical Treatment of Pulmonary Hydatid Disease in Children,” Medicinski Arhiv 61, no. 1 (2007): 11–15. 17582967

[3] D. A. Kıreşi , A. Karabacakoğlu , K. Ödev , and S. Karaköse , “Pictorial Review: Uncommon Locations of Hydatid Cysts,” Acta Radiologica 44, no. 6 (2003): 622–636. 14616207 10.1080/02841850312331287749

[4] K. Karavdic and S. Guska , “Surgical Treatement of Pulmonary Hydatid Disease in Children‐A Retrospective Study,” Medicinski Arhiv 65, no. 1 (2011): 16–19. 21534445

[5] M. Sarkar , R. Pathania , A. Jhobta , B. Thakur , and R. Chopra , “Cystic Pulmonary Hydatidosis,” Lung India: Official Organ of Indian Chest Society 33, no. 2 (2016): 179–191. 27051107 10.4103/0970-2113.177449 PMC4797438

[6] Y. Aydin , A. B. Ulas , A. G. Ahmed , and A. Eroglu , “Pulmonary Hydatid Cyst in Children and Adults: Diagnosis and Management,” Eurasian Journal of Medicine 54, no. 1 (2022): 133–144. 10.5152/eurasianjmed.2022.22289 PMC11163342 36655457

[7] M. H. T. Jalayeri , R. A. Sharifi far , N. Lashkarbolouk , and M. Mazandarani , “The Co‐Infection of Pulmonary Hydatid Cyst, Lophomoniasis and Tuberculosis in a Patient With Resistant Respiratory Symptoms; A Case Report Study,” BMC Infectious Diseases 24, no. 1 (2024): 11. 38166664 10.1186/s12879-023-08907-4 PMC10759524

[8] P. Moro and P. M. Schantz , “Echinococcosis: A Review,” International Journal of Infectious Diseases 13, no. 2 (2009): 125–133. 18938096 10.1016/j.ijid.2008.03.037

[9] A. Erdogan , A. Ayten , and A. Demircan , “Methods of Surgical Therapy in Pulmonary Hydatid Disease: Is Capitonnage Advantageous?,” ANZ Journal of Surgery 75, no. 11 (2005): 992–996. 16336395 10.1111/j.1445-2197.2005.03594.x

[10] M. A. Haberal , E. Akar , O. S. Dikis , and M. Kaya , “Surgical Treatment of Childhood Pulmonary Hydatidosis: An Analysis of 25 Cases,” Tanaffos 17, no. 4 (2018): 280–284. 31143219 PMC6534802

[11] T. He , X. Sun , Z. Zhang , B. Xu , and W. Liu , “Cystotomy With Non‐Capitonnage in Treating Children With Pulmonary Hydatid Disease,” Annals of Thoracic and Cardiovascular Surgery 28, no. 1 (2022): 41–47. 34321387 10.5761/atcs.oa.20-00390 PMC8915934

[12] E. H. Kabiri , M. El Hammoumi , and M. Kabiri , “Surgical Treatment of Hydatidothorax in Children: A Retrospective Study of 19 Patients,” Journal of Pediatric Surgery 55, no. 3 (2020): 433–436. 30929945 10.1016/j.jpedsurg.2019.03.003

[13] O. H. Kocaman , T. Günendi , O. Dere , M. E. Dörterler , and M. E. Boleken , “Pulmonary Hydatid Cyst in Children: A Single‐Institution Experience,” Cureus 14, no. 7 (2022): e26670. 35949804 10.7759/cureus.26670 PMC9357973

[14] K. Ngcobo , R. Madansein , M. Ndlovu , and R. Masekela , “Surgical Management of Pulmonary Hydatid Cysts in Children in Kwazulu‐Natal Province, South Africa,” African Journal of Thoracic and Critical Care Medicine 26, no. 3 (2020): 81–86. 10.7196/AJTCCM.2020.v26i3.108 PMC8203051 34240027

[15] M. J. Page , J. E. McKenzie , P. M. Bossuyt , et al., “The PRISMA 2020 Statement: An Updated Guideline for Reporting Systematic Reviews,” International Journal of Surgery 88 (2021): 105906. 33789826 10.1016/j.ijsu.2021.105906

[16] K. Knobloch , U. Yoon , and P. M. Vogt , “Preferred Reporting Items for Systematic Reviews and Meta‐Analyses (Prisma) Statement and Publication Bias,” Journal of Cranio‐Maxillofacial Surgery 39, no. 2 (2011): 91–92. 21145753 10.1016/j.jcms.2010.11.001

[17] S. S. Bahreiny , A. Ahangarpour , and M. Aghaei , “Circulating Levels of Advanced Glycation End Products in Females With Polycystic Ovary Syndrome: A Meta‐Analysis,” Reproductive and Developmental Medicine 8, no. 2 (2024): 93–100.

[18] A. H. Mahdizade , S. S. Bahreiny , M.‐N. Bastani , et al., “The Influence of CDKAL1 (rs7754840) Gene Polymorphism on Susceptibility to Gestational Diabetes Mellitus in Pregnant Women: A Systematic Review and Meta‐Analysis,” International Journal of Diabetes in Developing Countries 44, no. 1 (2024): 3–12.

[19] S. S. Bahreiny , M. Aghaei , M. R. Dabbagh , H. Ghorbani , M. Javidan , and R. M. Fard , “Exploring the Relationship Between Ambient Sulfur Dioxide and Semen Quality Parameters: A Systematic Review and Meta‐Analysis,” Asian Pacific Journal of Reproduction 13, no. 1 (2024): 12–21.

[20] M. Çevik , I. Eser , and M. E. Boleken , “Characteristics and Outcomes of Liver and Lung Hydatid Disease in Children,” Tropical Doctor 43, no. 3 (2013): 93–95. 23788277 10.1177/0049475513493415

[21] A. E. Balci , N. Eren , Ş. Eren , and R. Ülkü , “Ruptured Hydatid Cysts of the Lung in Children: Clinical Review and Results of Surgery,” Annals of Thoracic Surgery 74, no. 3 (2002): 889–892. 12238856 10.1016/s0003-4975(02)03785-2

[22] K. Amine , B. Samia , C. Jamila , et al., “Thoracoscopic Treatment of Pulmonary Hydatid Cyst in Children: A Report of 25 Cases,” La Tunisie Medicale 92, no. 5 (2014): 341–344. 25504388

[23] A. Yener , D. Aysenur , A. Omer , O. Hayri , U. Ali Bilal , and E. Atilla , “Pre‐School Children With Hydatid Disease of Lung,” European Respiratory Journal 40, no. Suppl 56 (2012): P2405.

[24] I. C. Kurkcuoglu , A. Eroglu , N. Karaoglanoglu , A. Turkyilmaz , C. Tekinbas , and A. Basoglu , “Surgical Approach of Pulmonary Hydatidosis in Childhood,” International Journal of Clinical Practice 59, no. 2 (2005): 168–172. 15854192 10.1111/j.1742-1241.2004.00275.x

[25] A. Ksia , M. B. Fredj , A. Zouaoui , et al., “Capitonnage Seems Better in Childhood Pulmonary Hydatid Cyst Surgery,” Journal of Pediatric Surgery 55, no. 4 (2020): 752–755. 31138449 10.1016/j.jpedsurg.2019.05.009

[26] A. Aqqad , B. Hamdi , S. Louhaichi , et al., “Giant Pulmonary Hydatid Cyst in Children,” Archives de Pédiatrie 28, no. 4 (2021): 273–277. 33773892 10.1016/j.arcped.2021.02.017

[27] R. DerSimonian and N. Laird , “Meta‐Analysis in Clinical Trials Revisited,” Contemporary Clinical Trials 45 (2015): 139–145. 26343745 10.1016/j.cct.2015.09.002 PMC4639420

[28] J. P. T. Higgins , “Measuring Inconsistency in Meta‐Analyses,” BMJ 327, no. 7414 (2003): 557–560. 12958120 10.1136/bmj.327.7414.557 PMC192859

[29] M. Egger , G. D. Smith , M. Schneider , and C. Minder , “Bias in Meta‐Analysis Detected by a Simple, Graphical Test,” BMJ 315, no. 7109 (1997): 629–634. 9310563 10.1136/bmj.315.7109.629 PMC2127453

[30] F. Sayir , U. Cobanoğlu , A. Sehitoğulları , and S. Bilici , “Our Eight‐Year Surgical Experience in Patients With Pulmonary Cyst Hydatid,” International Journal of Clinical and Experimental Medicine 5, no. 1 (2012): 64–71. 22328950 PMC3272688

[31] O. K. Salih , M. S. Topcuoğlu , S. K. Celik , T. Ulus , and A. Tokcan , “Surgical Treatment of Hydatid Cysts of the Lung: Analysis of 405 Patients,” Canadian Journal of Surgery 41, no. 2 (1998): 131–135. PMC3949826 9575996

[32] M. Çelik , C. Senol , M. Keles , et al., “Surgical Treatment of Pulmonary Hydatid Disease in Children: Report of 122 Cases,” Journal of Pediatric Surgery 35, no. 12 (2000): 1710–1713. 11101720 10.1053/jpsu.2000.19219

[33] A. Turna , M. A. Yılmaz , G. Hacıibrahimoğlu , C. Asım Kutlu , and M. A. Bedirhan , “Surgical Treatment of Pulmonary Hydatid Cysts: Is Capitonnage Necessary?,” Annals of Thoracic Surgery 74, no. 1 (2002): 191–195. 12118757 10.1016/s0003-4975(02)03643-3

[34] A. Şehitoğulları , “Our Results in Surgical Treatment of Hydatid Cyst of the Lungs,” European Journal of General Medicine 4, no. 1 (2007): 5–8.

[35] M. Aldahmashi , M. Alassal , I. Kasb , and H. Elrakhawy , “Conservative Surgical Management for Pulmonary Hydatid Cyst: Analysis and Outcome of 148 Cases,” Canadian Respiratory Journal 2016 (2016): 1–6. 10.1155/2016/8473070 PMC5013219 27642249

[36] I. Lichter , “Surgery of Pulmonary Hydatid Cyst—The Barrett Technique,” Thorax 27, no. 5 (1972): 529–534. 5083720 10.1136/thx.27.5.529 PMC470540

[37] E. Akar and M. Çakmak , “Surgical Therapy in Patients With Pulmonary Hydatidosis,” İzmir Göğüs Hastanesi Dergisi 28, no. 1 (2014): 9–13.

[38] S. Halezeroglu , M. Celik , A. Uysal , C. Senol , M. Keles , and B. Arman , “Giant Hydatid Cysts of the Lung,” Journal of Thoracic and Cardiovascular Surgery 113, no. 4 (1997): 712–717. 9104980 10.1016/S0022-5223(97)70228-9

[39] Y. Li , H. Zheng , X. Cao , Z. Liu , and L. Chen , “Demographic and Clinical Characteristics of Patients With Anaphylactic Shock After Surgery for Cystic Echinococcosis,” American Society of Tropical Medicine and Hygiene 85, no. 3 (2011): 452–455. 10.4269/ajtmh.2011.10-0448 PMC3163865 21896803

[40] K. Karavdić , B. Mehić , and S. Guska , “Presentation the New Surgicaly Approach in the Treatment of Pediatric Lung Hydatid Disease Non–Captonage Procedure Without Closure of the Communication Bronchial Opening,” J Lung Pulm Respir Res 6, no. 1 (2019): 7–15.

[41] C. Akgul Ozmen and S. Onat , “Computed Tomography (CT) Findings of Pulmonary Hydatid Cysts in Children and the Factors Related to Cyst Rupture,” Medical Science Monitor 23 (2017): 3679–3686. 28754885 10.12659/MSM.906163 PMC5546193

[42] M. Dakak , H. Caylak , K. Kavakli , et al., “Parenchyma‐Saving Surgical Treatment of Giant Pulmonary Hydatid Cysts,” Thoracic and Cardiovascular Surgeon 57, no. 03 (2009): 165–168. 19330755 10.1055/s-2008-1039210

[43] O. Usluer , K. C. Ceylan , S. Kaya , S. Sevinc , and S. Gursoy , “Surgical Management of Pulmonary Hydatid Cysts: Is Size an Important Prognostic Indicator?,” Texas Heart Institute Journal 37, no. 4 (2010): 429–434. 20844615 PMC2929855

[44] A. Kuzucu , H. Ulutas , M. Reha Celik , and E. Yekeler , “Hydatid Cysts of the Lung: Lesion Size in Relation to Clinical Presentation and Therapeutic Approach,” Surgery Today 44 (2014): 131–136. 23334707 10.1007/s00595-012-0484-2

[45] J. Eckert , “Guidelines for Treatment of Cystic and Alveolar Echinococcosis in Humans,” Bulletin of the World Health Organization 74, no. 3 (1996): 231–242. 8789923 PMC2486920

[46] B. Aydogdu , S. Sander , O. Demirali , et al., “Treatment of Spontaneous Rupture of Lung Hydatid Cysts Into a Bronchus in Children,” Journal of Pediatric Surgery 50, no. 9 (2015): 1481–1483. 25783398 10.1016/j.jpedsurg.2015.01.010

[47] N. Karaoglanoglu , I. C. Kurkcuoglu , M. Gorguner , A. Eroglu , and A. Turkyilmaz , “Giant Hydatid Lung Cysts,” European Journal of Cardio‐Thoracic Surgery 19, no. 6 (2001): 914–917. 11404152 10.1016/s1010-7940(01)00687-x

